# US Black Maternal Health Advocacy Topics and Trends on Twitter: Temporal Infoveillance Study

**DOI:** 10.2196/30885

**Published:** 2022-04-20

**Authors:** Diana Grigsby-Toussaint, Ashley Champagne, Justin Uhr, Elizabeth Silva, Madeline Noh, Adam Bradley, Patrick Rashleigh

**Affiliations:** 1 Department of Epidemiology School of Public Health Brown University Providence, RI United States; 2 Department of Behavioral and Social Sciences School of Public Health Brown University Providence, RI United States; 3 Brown University Library Providence, RI United States; 4 Department of Anthropology School of Public Health Brown University Providence, RI United States

**Keywords:** Black maternal health, disparity, COVID-19, Twitter, topic modeling, digital humanities, infoveillance, maternal health, minority, women, advocacy, social media, model, trend, feasibility

## Abstract

**Background:**

Black women in the United States disproportionately suffer adverse pregnancy and birth outcomes compared to White women. Economic adversity and implicit bias during clinical encounters may lead to physiological responses that place Black women at higher risk for adverse birth outcomes. The novel coronavirus disease of 2019 (COVID-19) further exacerbated this risk, as safety protocols increased social isolation in clinical settings, thereby limiting opportunities to advocate for unbiased care. Twitter, 1 of the most popular social networking sites, has been used to study a variety of issues of public interest, including health care. This study considers whether posts on Twitter accurately reflect public discourse during the COVID-19 pandemic and are being used in infodemiology studies by public health experts.

**Objective:**

This study aims to assess the feasibility of Twitter for identifying public discourse related to social determinants of health and advocacy that influence maternal health among Black women across the United States and to examine trends in sentiment between 2019 and 2020 in the context of the COVID-19 pandemic.

**Methods:**

Tweets were collected from March 1 to July 13, 2020, from 21 organizations and influencers and from 4 hashtags that focused on Black maternal health. Additionally, tweets from the same organizations and hashtags were collected from the year prior, from March 1 to July 13, 2019. Twint, a Python programming library, was used for data collection and analysis. We gathered the text of approximately 17,000 tweets, as well as all publicly available metadata. Topic modeling and k-means clustering were used to analyze the tweets.

**Results:**

A variety of trends were observed when comparing the 2020 data set to the 2019 data set from the same period. The percentages listed for each topic are probabilities of that topic occurring in our corpus. In our topic models, tweets on reproductive justice, maternal mortality crises, and patient care increased by 67.46% in 2020 versus 2019. Topics on community, advocacy, and health equity increased by over 30% in 2020 versus 2019. In contrast, tweet topics that decreased in 2020 versus 2019 were as follows: tweets on Medicaid and medical coverage decreased by 27.73%, and discussions about creating space for Black women decreased by just under 30%.

**Conclusions:**

The results indicate that the COVID-19 pandemic may have spurred an increased focus on advocating for improved reproductive health and maternal health outcomes among Black women in the United States. Further analyses are needed to capture a longer time frame that encompasses more of the pandemic, as well as more diverse voices to confirm the robustness of the findings. We also concluded that Twitter is an effective source for providing a snapshot of relevant topics to guide Black maternal health advocacy efforts.

## Introduction

Compared to White women, Black women are 3 times more likely to have pregnancy-related deaths in the United States (13 deaths per 100,000 births compared to 41 deaths per 100,000 births) [1]. Black infants also die at twice the rate of White infants (10.8 deaths per 1000 compared to 4.6 deaths per 1000) [2]. The COVID-19 pandemic, combined with endemic vulnerabilities of structural racism and biased care, has further exacerbated these disparities. Blacks are disproportionately impacted by COVID-19, dying at 3 times the rate of Whites [3], and in some cities, pregnant Black women were found to be 5 times more likely to be exposed to COVID-19 compared to pregnant White women [4]. As evidenced by several articles in the popular press, Black women continue to experience biased care during the pandemic [5]. Black women have more risk factors (eg, obesity) for COVID-19 and are more likely to work in occupations (eg, nurses’ aides) that increase exposure to COVID-19 [6]. Policies that were implemented to reduce the spread of COVID-19 (eg, increased use of telemedicine for patient visits, separation of mothers from newborns) may further place Black mothers at increased risk due to increased social isolation [1,7]. Additionally, structural racism, as evidenced by acts of police violence against Blacks, have continued since the beginning of the pandemic [8].

Persistent poor reproductive and birth outcomes among Black women precipitated the introduction of H.R. 6142, the Black Maternal Health Momnibus Act of 2021, by members of the US Congress in July 2020 [9]. The bill seeks to address social factors driving the Black maternal health crisis in the United States, such as housing, nutrition, and access to culturally responsive care, in addition to supporting robust metrics to evaluate impact. The Black maternal health crisis is so entrenched in the United States, however, that several states have also sought various legislative avenues for amelioration while federal efforts play out. In Illinois, for example, House Bill 1, which created a Task Force on Infant and Maternal Mortality Among African Americans, was passed in July 2019 [10]. In January 2020, Illinois passed House Bill 2, which includes additional rights for pregnant women as part of the Medical Patients’ Rights Act, including “the right to be treated with respect at all times before, during, and after pregnancy by [...] health care professionals and to have a health care professional that is culturally competent and treats her appropriately regardless of her ethnicity, sexual orientation, or religious background” [11]. California, cognizant that the observed racial disparities in maternal and birth outcomes cannot be entirely explained by education or access to prenatal care, passed Senate Bill 464, the California Dignity in Pregnancy and Childbirth Act, in 2019 [12]. In addition to tracking and publishing data on maternal mortality rates, the legislation also requires implicit bias training for all perinatal health care providers. The hope is that providers will learn to recognize their unconscious prejudices or stereotypes in their interactions with Black and other minoritized women, resulting in more empathetic care that reduces adverse pregnancy and birth outcomes. The importance of the aforementioned legislative efforts around the Black maternal health crisis have clearly been amplified by the COVID-19 pandemic, with recent exhortations from maternal and child health experts to develop policies to immediately and effectively address this crisis [13].

Social media offers an important window into public discourse on maternal and birth outcomes, and our study looks particularly at Twitter. Twitter is 1 of the most popular social networking sites, with 192 million daily active users and approximately 500 million tweets shared per day [14]. It has been used to study a variety of issues of public interest, including health care and mental health, among others [15]. Although approximately 9% of Black US adults indicate noninternet usage [16], a recent study found that racial and ethnic minority groups were more likely to post COVID-19-related content on social media [17]. Moreover, Twitter is considered a social media platform that may accurately capture public discourse during the COVID-19 pandemic and is being used in several infodemiology studies by public health experts [17]. As such, we found Twitter to be an appropriate platform to examine public discourse from Black maternal health organizations and influencers on Twitter within the context of COVID-19.

In this paper, we are particularly interested in the impact of COVID-19 on advocacy issues for Black maternal health and whether advocacy efforts have changed or remained the same as a consequence of the pandemic. Specifically, we are interested in understanding public discourse related to social determinants of health and advocacy that influence maternal health among Black women in the United States and examining topics and trends in sentiment between 2019 and 2020 in the context of the COVID-19 pandemic. We hypothesize that there will be an increase in tweets related to advocacy efforts for Black women, as the COVID-19 pandemic has exacerbated existing disparities in maternal and child health in this group.

## Methods

### Data Collection

Tweets were collected from March 1 to July 13 for 2019 and 2020 from 21 organizations and influencers and from 4 hashtags that focused on Black maternal health. Twint, a Python programming library, was used for data collection and analyses [15]. We gathered the texts of approximately 17,000 tweets, as well as all publicly available metadata. Topic modeling and k-means clustering were used to analyze the tweets.

To gather relevant tweets for analysis, we researched organizations and influencers who are focused on supporting Black maternal health. We also identified hashtags that people often used to communicate about Black maternal health. We curated a list of accounts, in part, by researching organizations that supported the Black Maternal Health Momnibus Act of 2021 [9]. Second, we identified which organizations in that list had active Twitter accounts. Our criteria for “active user” included regular tweets posted throughout the 2 time periods we wanted to collect material: March 1-July 13 in both 2019 and 2020. We wanted to gather tweets that were shared from these organizations and influencers during the early period of the pandemic and compare those tweets with the same period the year prior to the pandemic. We started collecting tweets on March 1, a week or two before most cities in the United States shut down, because the US Centers for Disease Control and Prevention (CDC) concluded that COVID-19 was heading toward pandemic status even before lockdowns began [18]. Although not every tweet gathered contained the word “COVID” or “pandemic,” each tweet collected within the 2020 data set was shared during the pandemic. By gathering both a data set from 2020 and from the year prior, we can start to understand how the messaging from advocates of Black maternal health changed during the pandemic to support Black women and families.

The study was deemed IRB-exempt due to the use of publicly available Twitter data that was anonymized.

We gathered the text of the tweets, as well as all publicly available metadata from organizations, influencers, and hashtags that advocate for Black maternal health. They are (with the exclusion of names of personal accounts) listed in Table 1.

**Table 1:** Twitter accounts, hashtags, and geographic locations.

Twitter account or hashtag	Location of organization, if available
Black Mamas Matter Alliance (BlkMamasMatter)	—^a^
Black Women’s Health (blkwomenshealth)	Washington, DC
National Birth Equity Collab (BirthEquity)	New Orleans, LA
In Our Own Voice (BlackWomensRJ)	Washington, DC
Sister Reach (SisterReach)	Memphis, TN
Sister Song (SisterSong_WOC)	Atlanta, GA
MS Black Women’s Roundtable (msblackwomensr1)	Jackson, MS
Moms Rising (MomsRising)	United States
Shades of Blue (shadesofblueprj)	Houston, TX
Mount Sinai Health System (MountSinaiWHRI)	—
Black Maternal Health Caucus (BMHCaucus)	Washington, DC
Mama Glow (MamaGlow_MGFF)	New York City, NY; Los Angeles, CA; Miami, FL; Paris, France
The National Association to Advance Black Birth (thenaabb)	Washington, DC
Balanced Black Girl (balancedblkgirl)	Los Angeles, CA
California Black Women’s Health Project (cabwhp)	Inglewood, CA
The Frugal Feminista (frugalfeminista)	New York
JOY Collective (aJOYcollective)	United States
Abiola Abrams (abiolatv)	—
Loretta J. Ross (lorettajross)	Atlanta, GA
Linda Goler Blount (lindagblount)	Washington, DC
Dr. Joia Crear-Perry (doccrearperry)	New Orleans, LA
#blackmaternalmortality	N/A^b^
#blackmaternalhealth	N/A
#bwwday	N/A

^a^Not available.

^b^N/A: not applicable.

We included the hashtags #blackmaternalmortality, #blackmaternalhealth, and #bwwday as they are popular hashtags that capture general content about Black maternal health and wellness.

Although we originally sought out to collect tweets by searching for mentions of text such as “Black maternal health” and “Black women” and discussions around pregnancy complications, our resulting data set was not as focused as we wanted it to be on Black maternal health. Specifically, searching for phrases on Twitter gathers tweets that are not on Black maternal health but contain the phrase “Black women.” Gathering tweets from organizations, in contrast, and hashtags that are specific enough about Black maternal health produces a data set that is more specific to Black maternal health. Although we could have “cleaned” the data set to omit tweets that did not make sense to include because they were not about Black maternal health, such cleaning would have added bias to the data set as the choices about what to include or not would have been determined by the authors. Thus, we focused our data set on organizations and a few specific hashtags to gather a sample data set on Black maternal health.

Although we set our parameters for data collection so that retweets were not included, the texts and hashtags of all other tweets were gathered from the accounts, influencers, and hashtags we selected. Our tweets did include “quoted tweets” or tweets that cited another user and shared what they wrote but without retweeting them. Although the existence of quoted tweets in our data set introduced some bias as it potentially amplified the text of a given tweet, retweets were not a large portion of the data set.

To analyze the tweets, we used 2 methods: topic modeling and k-means clustering. We found that topic modeling yielded the most useful results, and those are described next. Notable results from k-means clustering are available in Multimedia Appendix 1.

Topic modeling attempts to detect groups of words that occur together frequently in the same document. In our case, each tweet was a document. In topic modeling, the “topics” are composed of words within the documents as a whole that co-occur; they are not necessarily words or phrases that a human might use to summarize a topic. It is common practice within the digital humanities to produce human labels to describe the topics [19]. We worked in pairs to determine labels for each topic using an iterative process. Each reviewer first examined the topics independently to determine a label and then met with the second reviewer to reach agreement on the final labels assigned.

Our data preprocessing steps were as follows. We merged the tabular data from 2019 and 2020 into a single Pandas Python Data Analysis Library DataFrame, retaining the tweets themselves along with the year portion of the date [20]. We extracted the tweets from the DataFrame into a list. Then, we cleaned the data by removing uniform resource locators (URLs), newlines, and apostrophes. We also temporarily removed “@” tags to prevent them from being modified by other steps in the preprocessing. We then used the Gensim (RARE Technologies Ltd) built-in simple preprocess function to further clean the text and convert each tweet from a string into a list of lowercase words [21]. We largely used the default parameters, except that we converted accented characters to their unaccented equivalents. We removed the default Natural Language Toolkit (NLTK) English stop words [22]. We cleaned our tweets in this way because we wanted the algorithm to read the words as close to their context as possible. So, for example, had we not changed the words to lowercase, the algorithm would have seen “Dance” as different from “dance” and counted them as separate. Cleaning the text in this way allows researchers to identify how the words co-occur in the tweets without considering capitalization.

We then wanted to make sure our analysis could differentiate between phrases and individual words. For example, we did not want to count the word “three” in “The Three Musketeers” the same as the word “three” in other contexts. So, we then used the Gensim *Phrases* function to combine words that commonly occurred together into word compounds [21]. This was done twice to join together phrases with more than 2 words. Then, we lemmatized the words and filtered out words that were not nouns, adjectives, verbs, adverbs, or proper nouns. Adding the hashtags and “@” tags back in at this point allowed us to later analyze the tweets by hashtag or mention. Finally, we removed words that occurred only once, and removed any word lists that were blank as a result of performing the previous steps. In addition, we converted the word lists to bag-of-words model ID and frequency pairs.

To create our topic models, we used Gensim’s Latent Dirichlet Allocation (LDA) model [21]. We set the number of topics to 109 because that is where we noticed a peak of the coherence score at 0.5318. Above this number, the score initially decreased. Although the score did eventually begin to increase again with more topics, even with several hundred topics the score remained below this peak. In addition, based on our human readings of the topics, 109 topics generated the most coherent models. We determined that analysis would become unwieldy beyond a few hundred topics, and therefore, it would not be worth increasing the number of topics further in search of a higher score.

We set the *random state* parameter to 100 arbitrarily. We set the number of passes to 10. We set the *alpha* parameter to “auto.” All other parameters used the default value. For each topic, we calculated its composition of tweets from 2019 to 2020 and used this to determine which topics increased or decreased in significance between the 2 time periods.

## Results

### Trends Observed

We saw a variety of trends when we analyzed 17,000 tweets in our corpus and compared the 2020 data set to the 2019 data set from the same period. Based on the results of the topic models, tweets on reproductive justice, maternal mortality crisis, and patient care increased by over 65% in 2020 versus 2019. Topics on community, advocacy, and health equity increased by over 30% in 2020 versus 2019. In contrast, tweet topics that decreased in 2020 versus 2019 included tweets on Medicaid and medical coverage, which decreased by 27.73%, and discussions about creating space for Black women, which decreased by just under 30%. This change in what Black maternal health activists discussed on Twitter indicates a shift in their concerns from Medicaid and medical coverage to reproductive justice, the maternal mortality crisis, and health equity more broadly.

Our results indicate that the COVID-19 pandemic may have spurred an increased focus on advocating for improved reproductive health and maternal health outcomes among Black women in the United States. Although the terms “COVID” and “pandemic” are not grouped into 1 topic in the 2020 data set, all of the tweets within this data set were shared during the early stages of the pandemic and, therefore, speak to the messaging by Black maternal health organizations and advocates during the COVID-19 pandemic. All the content of the tweets from both the 2019 and 2020 data sets was included in the topic models; we then analyzed the results to understand how messaging had shifted during the pandemic. We manually annotated the tweets that were correlated with the topics outputted by LDA. Further analyses are needed to capture a longer time frame that encompasses more of the pandemic, as well as additional analysis of messaging by Black maternal health advocates on other platforms to confirm the robustness of our findings.

**Figure 1:**
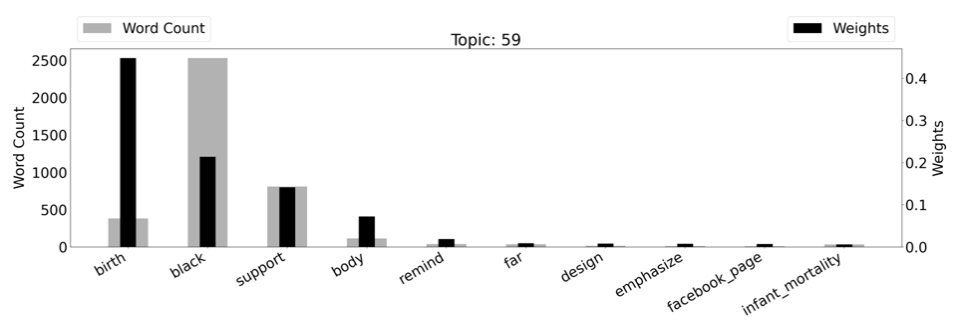
Topic 59 (“Reproductive Justice”) with weighted word counts.

A sample of the topic models is available in Multimedia Appendix 1. The percentages listed for each word are probabilities of that word occurring in the given topic. As an example, the top words within the reproductive justice topic model were birth (45% of the topic), black (21% of the topic), support (14% of the topic), and body (7% of the topic). The words within each topic model were both weighted and counted. For words that appeared frequently within our corpus, such as “black,” the word had a lower weight value but a high word count. In contrast, a word like “birth” had a high weight value but a low word count value. Each weight and word count were determined using TfidfVectorizer (Sklearn). Figure 1 highlights the weighted word counts for one of our topics, “Reproductive Justice.”

The “Word Count” listed in the chart refers to the number of times each respective word appeared in the text. For example, the word “remind” appeared less than 500 times. The “weight” of a word refers to how common the word is in association to the rest of the corpus. The less common the word, the higher the weight of the word. So, for example, the word “birth” had a high word count but a low weight; this is because the word “birth” appeared so frequently in our corpus that it was less significant when the word appeared. However, the word “black,” had a lower word count and a higher weight as it appeared less frequently in our data set compared to the rest of the corpus. A word cloud visualizing topic 59 can also be found in Multimedia Appendix 2.

Here is an example tweet associated with this topic:

Advocating for the rights of Black birthing people is always important, but even more so in the midst of the COVID19 pandemic. The National Association to Advance Black Birth- NAABB just launched a bill of rights for Black birthing people: https://thenaabb.org/index.php/black-birthing-bill-of-| rights/ …. #BMHW20 pic.twitter.com/5c8PhtwQQY

**Figure 2:**
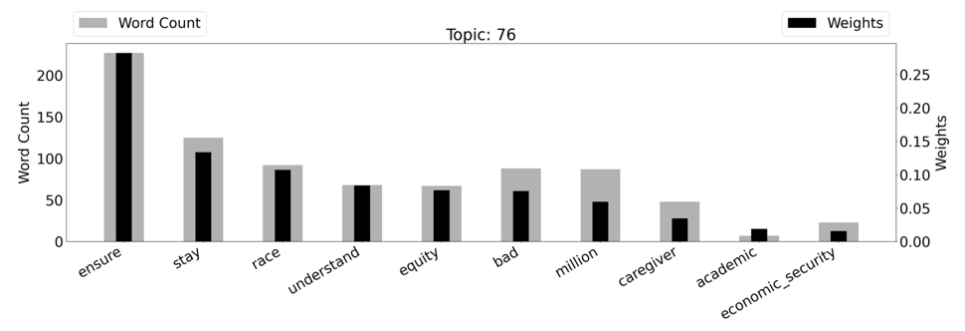
Topic 76 (“Advocacy”) with weighted word counts.

The topic model on advocacy showed a 33.3% increase in prevalence in 2020 versus 2019. The graph in Figure 2 displays the weights and word counts for top words within the topic.

We manually annotated the topic “Advocacy” as the majority of documents that make up this topic are related to equity in health. The term “ensure” is often used within the context of ensuring equity. For example, 1 tweet asks, “How do we ensure that minorities are no longer underrepresented in precision medicine? #SaludTues.” The hashtag #SaludTues is a monthly Tweetchat on Latino health hosted by the Institute for Health Promotion Research (IHPR) at University of Texas Health at San Antonio, which directs *Salud America!*

To understand the topic models produced by the LDA algorithm, we found it essential to combine manual reading of the tweets that most heavily make up a given topic with the quantitative results of the LDA algorithm. This is perhaps especially important when reviewing a largely general topic, such as this one. A word cloud visualizing topic 76 can also be found in Multimedia Appendix 3.

**Figure 3:**
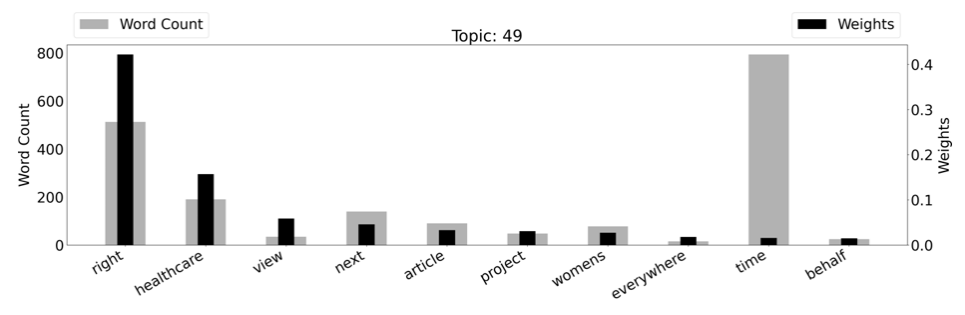
Topic 49 (“Health Equity”) with weighted word counts.

Another tweet topic that increased by 31.53% in 2020 versus 2019 centered on health equity. This topic (assigned topic 49) included stories ranging from C-section problems to celebrations of midwives and doulas to posts advocating for policies for maternal health. Figure 3 is a graph of the weighted word counts and top words within this topic.

Topic 49, in contrast to topic 76, was more focused: this topic focused on equity and rights within health care. The word “right” that appeared so heavily both in the word count and as a weighted word, appeared in tweets advocating for the rights of Black women that have historically been, and still are, neglected in health care. A word cloud visualizing topic 49 can also be found in Multimedia Appendix 4.

Here are some example tweets within this topic on health equity:

I had “fluid overload” from the c-section and was drowning . . .There’s warnings everywhere saying you can experience this after a c-section, and no one at the hospital told me. #blackmaternalhealth https://twitter.com/Essence/status/1103766054566805504 …

Happy #InternationalDayoftheMidwife! We salute and honor the historical contributions and traditions of #BlackMidwives and #BlackBirthWorkers on the front lines of #BlackMaternalHealth. #BlackMamasMatter pic.twitter.com/waA67xNBJM

In contrast, in 2020 versus 2019, tweet topics on Medicaid and medical coverage decreased by 27.73% and discussions about creating space for Black women decreased by just under 30%.

**Figure 4:**
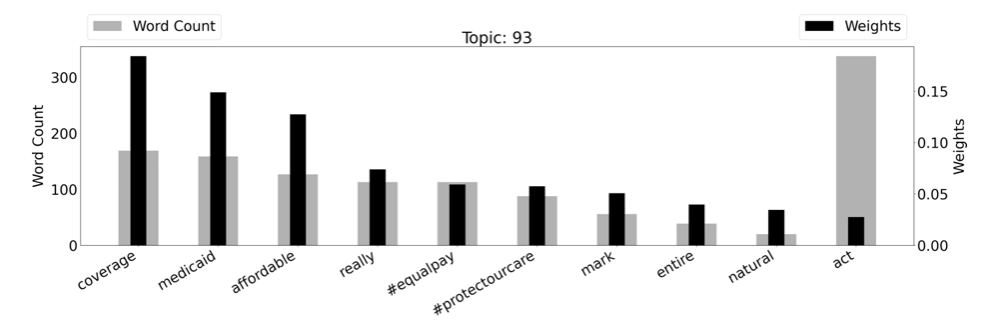
Topic 93 (“Medicaid and Medical Coverage”) with weighted word counts.

Topic 93, which focuses on Medicaid and medical coverage, included tweets about protecting care, the Affordable Care Act, and equal pay. The topic was focused; the words “coverage,” “medicaid,” and affordable” and the hashtag “#protectourcare” featured heavily. Figure 4 is a graph of the weighted word counts and top words within this topic. Topic 93 is also visualized in a word cloud in Multimedia Appendix 5.

Several relevant tweet examples that were included in this topic are as follows:

MOMMIES Act Seeks To Expand Medicaid Coverage For Pregnant Women

https://www.essence.com/news/mommies-act-cory-booker-​ayanna-pressley-medicaid/?utm_source=twitter.com&utm_​medium=social&utm_campaign=social-button-sharing … via @ESSENCE #MaternalJustice

#PaycheckFairness Act is part of the solution for #EqualPay, but we also need #paidsickdays,

#paidleave, affordable #childcare & #raisethewage to close the wage gap. #EqualPayDay

The decline in how much Black maternal health advocates talked about Medicaid and coverage in 2020 versus 2019 suggests that the topic was of more central importance before the pandemic. As the pandemic began, Black maternal health advocates began focusing more on health equity and advocacy more broadly.

**Figure 5:**
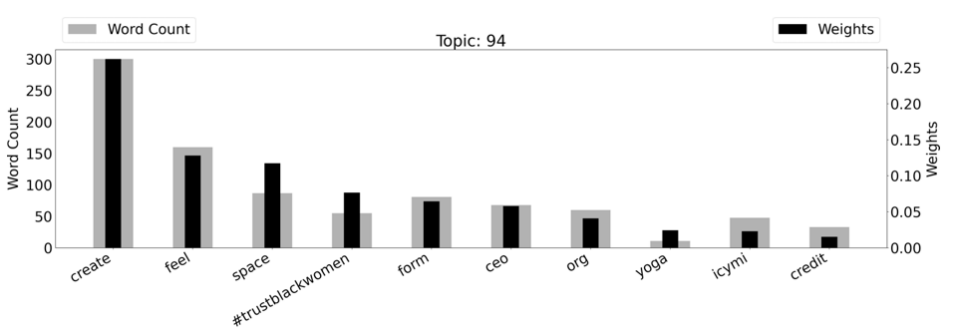
Topic 94 (“Creating Space”) with weighted word counts.

The “Creating Space” topic included discussions around giving Black women credit for the work they do, creating inclusive spaces, and trusting Black women. This topic decreased by almost 30% in 2020 compared to the same period in 2019. Figure 5 is a graph of the weighted word counts and top words within this topic. Topic 94 is also visualized in a word cloud in Multimedia Appendix 6.

A few example tweets for this topic include:

We're live! #MissionHearHer #TrustBlackWomen #StandWithBlackWomen @missionprtnrs https://www.facebook.com/BlackWomensRJ/videos/​409856806524798/ …

#BlackMaternalHealth Policy should center solutions with supporting resources for those actually doing the work in black communities across the nation, including our Kindred Partner members! #BMHCSummit #BMHCaucus #BlackMamasMatter #TrustBlackWomen #MaternalJustice https://twitter.com/BlkMamasMatter/status/​1118650545869393922

Due to the decline in Black maternal health advocates sharing information about supporting and trusting Black women, this topic suggests that Black maternal health advocates need to focus more on health equity within health care systems and reproductive justice. Because our topic models were created with the same corpus, over 20,000 tweets in total, each topic included both the 2019 data set and the 2020 data set. Tweets were removed when they appeared from accounts that were only active in 2019 versus 2020 or vice versa, as our goal was to understand how discourse changed, if at all, in the early pandemic months versus the same set of time within 2019, before the pandemic. Thus, our results show that there was an increase in tweets about reproductive justice and advocacy and a significant decrease in conversations on medical coverage and Medicaid; there was also a significant decrease in posts devoted to trusting Black women and creating inclusive spaces, although that support for Black women was focused on other solutions, such as economic policies (eg, paid family leave and support for Black women’s bodies).

## Discussion

### Principal Findings

Our findings are consistent with previous studies showing the importance of using Twitter to capture authentic expressions of experiences with health care and other aspects of life by minoritized groups in the United States [23] and the increased use of Twitter by Blacks [15]. We found this particular social media platform useful for assessing public discourse around Black maternal health issues in the context of COVID-19.

The discourse we studied on Twitter is congruent with national and local efforts that align with the US Department of Health and Human Services’ objective of reducing maternal mortality by 50% in the next 5 years [24]. Specific examples of recent legislative efforts include S.916/ H.R. 1897, the Mothers and Offspring Mortality and Morbidity Awareness Act (the MOMMA’s Act), re-introduced by Congresswoman Robin D. Kelly from Illinois to the 117th Congress [25]. The MOMMA’s Act seeks to improve and standardize reporting on maternal health care issues, in addition to reducing implicit bias and improving postpartum care. The Connected Maternal Online Monitoring Act -Mom Act (S.801) would protect the bodies of all mothers through remote monitoring of physiologic processes, such as blood pressure and blood glucose, as part of an expansion of telehealth efforts for pregnant and postpartum women [26]. In addition, the Family and Medical Insurance Leave (FAMILY Act) would result in a national insurance fund to cover 12 weeks per year to support the postpartum period as well as other health conditions [27]. Specific policies that would be helpful for Black mothers are being developed or waiting for movement in Congress or state legislatures. Those efforts, and their heightened importance due to COVID-19, are reflected in our results concerning advocacy and health equity.

We were less likely, however, to find legislation that focused specifically on the importance of having Black women at the forefront of efforts to ensure maternal justice exists. This is clearly a critical area of advocacy for Black maternal health in the United States, as only 5% of physicians are Black [28]. Moreover, there is some evidence to suggest that Black babies are more likely to thrive when they are cared for by Black physicians [29]. However, the extant literature highlights implicit bias in prenatal and postpartum care, as noted by 1 representative tweet:

There’s a lot of interest in health equity, without an understanding of what health equity is. Let's fix that!

Anyone who is interested in addressing the Black maternal health crisis in the United States must also gain a true understanding of the inequities that lead to the disparities between Black women and other racial and ethnic groups. This study highlights the importance of that research.

Our analysis is also important for showing the utility of Twitter as a platform for gaining insight into Black maternal health issues both in terms of messaging and as a tool for future advocacy efforts. First, a recent analysis by the Pew Research Center found that Blacks (45%) are more likely to use Twitter for political activism, such as “encouraging others to take action about issues important to them,” compared to Whites (30%) and Hispanics (33%) [30]. Consequently, although a higher percentage of US adults use Facebook (69%) and YouTube (81%) compared to Twitter (23%), Blacks are more likely to not only use Twitter (29%) but also use it to advocate for political and social issues [30,31]. Additionally, increased use of Twitter for advocacy has been tied to recent current events of concern among Blacks in the United States, such the killing of unarmed Black men (eg, George Floyd) [30,31]. Twitter has also been the social media platform of choice to advocate for #AmberIsaac, a Black woman who died following childbirth after high-risk symptoms were possibly missed due to COVID-19 restrictions on in-person prenatal care visits [32]. Thus, our use of Twitter to examine public discourse around legislative and policy efforts supporting Black maternal health in the United States is warranted by the literature. Notwithstanding, our analysis showed that Twitter is used primarily to share and amplify messages but less for articulating specific steps to move legislation forward. For example, although members of Congress [33] have some presence on Twitter and other social media platforms, few tweets specifically encouraged contacting or engaging members of Congress about advocating for specific policies or legislation. Future studies could use findings from Twitter content on advocacy to engage in more explicit efforts to push for policy changes, in addition to sharing messages or information about events of interest.

During a period with limited opportunity for primary data collection, Twitter served as a tool for identifying organizations engaged in advocacy efforts for Black women, and the topics identified were aligned with the extant literature, providing a timely snapshot for areas of focus. Future work could also use Twitter to identify issues of importance for Black maternal health and use the platform to garner support for specific legislative efforts and policies at federal, state, and local levels.

### Limitations

As with any social media platform, Twitter has population bias. A study by Ruths and Pfeffer [34] noted that there are sampling biases in every social media platform: “Instagram is ‘especially appealing to adults aged 18-29, African-American, Latinos, women, urban residents’ whereas Pinterest is dominated by females, age between 25-34, with an average annual household income of $100,000” [34]. The Pew Research Center notes that Twitter users tend to be younger and have higher incomes than people in the United States overall, although the race and ethnicity of Twitter users largely mirrors that of all US adults [35]. Additionally, it is important to note that the tweets we analyzed come from a specific subset of Twitter users who are primarily Black women involved in advocacy efforts for Black maternal health. Thus, although Twitter has population bias, we gathered tweets specifically by Black women and organizations in support of Black women in order to yield a relevant data set for our study. Additionally, Jules et al [36] note ethical issues in collecting Twitter data, 1 of which is that users have not necessarily given informed consent for researchers to gather their tweets and analyze them [36]. In response, we anonymized our data set to protect users.

It is also important to note that the results of this analysis are not generalizable due to the small sample size of posts reviewed (approximately 10%). As such, our results are mostly exploratory and should be followed up with further study.

### Conclusion

The disparities present in maternal mortality between Black and White women have persisted for the past 100 years [37]. Non-Hispanic Black women suffer from the highest rates of 22 (88%) of 25 severe maternal morbidity indicators, according to the CDC [37], and non-Hispanic Black infants have the highest rates of infant mortality and preterm birth in the United States, being more than twice as likely to die during their first year of life compared with their White counterparts [38]. The entrenched US Black maternal and infant health crisis has been heightened due to the disproportionate impact of COVID-19 on Blacks and other minoritized groups, with higher prevalence and mortality rates due to SARS-CoV-2 compared to Whites [7]. Prior to the pandemic, several efforts were underway at the federal, state, and local levels to address the maternal and infant health crisis [11-14]. However, at the time that this study was conducted, there was no other systematic social media analysis of how Black maternal health advocacy issues were impacted by the pandemic. In our Twitter analysis, we found that discussion of issues of reproductive justice, equity, and advocacy increased considerably between 2019 and 2020. The high presence of these important issues in our topic models further confirms the ongoing nature of the Black maternal health crisis. Interestingly, issues around health care coverage, such as Medicaid or medical coverage in general, decreased, which may be due to the possibility that simply having access to care does not eliminate adverse maternal and birth outcomes for Black Americans. Rather, addressing issues around implicit bias and social determinants of health may play a greater role in mitigating the Black maternal health crisis. Our analysis is important for thinking about effective national policies that may improve the long-term health and safety of Black women and their children.

## Appendix

Multimedia Appendix 1 Topic models and results from k-means clustering.

Multimedia Appendix 2 Topic 59 (“Reproductive Justice”) visualized in a word cloud.

Multimedia Appendix 3 Topic 76 (“Advocacy”) visualized in a word cloud.

Multimedia Appendix 4 Topic 49 (“Health Equity”) visualized in a word cloud.

Multimedia Appendix 5 Topic 93 (“Medicaid and Medical Coverage”) word cloud.

Multimedia Appendix 6 Topic 94 (“Creating Space”) visualized in a word cloud.

## Abbreviations

CDC: Centers for Disease Control and Prevention
MOMMA’s Act: Mothers and Offspring Mortality and Morbidity Awareness Act
