# Supplementary material for: US Black Maternal Health Advocacy Topics and Trends on Twitter: Temporal Infoveillance Study
Source: JMIR Infodemiology. 2022 Apr 20;2(1):e30885. doi: 10.2196/30885 (PMC9092478; doi:10.2196/30885)

**Multimedia Appendix 1. Topic models and results from k-means clustering.**

**Topic models**

The topic models are available below along with the percentage the topic increased or decreased in 2020 versus 2019.

**Topic #59:**

**45% | "birth"**

**21% | "black"**

**14% | "support"**

**07% | "body"**

**2019 Percentage: 33.18% 2020 Percentage: 66.82% Percentage Increase: 101.38%**

**____________________________________**

**Topic #28:**

**31% | "crisis"**

**13% | "maternal_mortality"**

**09% | "movement"**

**09% | "click"**

**08% | "racial"**

**05% | "saturday"**

**2019 Percentage: 37.39% 2020 Percentage: 62.61% Percentage Increase: 67.46%**

**____________________________________**

**Topic #31:**

**16% | "critical"**

**11% | "strong"**

**10% | "doctor"**

**06% | "nurse"**

**06% | "treatment"**

**05% | "@youtube"**

**2019 Percentage: 37.67% 2020 Percentage: 62.33% Percentage Increase: 65.44%**

**____________________________________**

**Topic #86:**

**25% | "ever"**

**16% | "especially"**

**14% | "lose"**

**08% | "email"**

**2019 Percentage: 37.77% 2020 Percentage: 62.23% Percentage Increase: 64.74%**

**____________________________________**

**Topic #71:**

**31% | "link"**

**06% | "write"**

**06% | "will"**

**06% | "hit"**

**06% | "instead"**

**2019 Percentage: 37.79% 2020 Percentage: 62.21% Percentage Increase: 64.59%**

**____________________________________**

**Topic #103:**

**26% | "even"**

**25% | "series"**

**08% | "folk"**

**08% | "close"**

**2019 Percentage: 40.23% 2020 Percentage: 59.77% Percentage Increase: 48.59%**

**____________________________________**

**Topic #90:**

**22% | "make_sure"**

**15% | "post"**

**06% | "rt"**

**06% | "identify"**

**05% | "ignore"**

**05% | "deliver"**

**2019 Percentage: 40.49% 2020 Percentage: 59.51% Percentage Increase: 46.99%**

**____________________________________**

**Topic #4 :**

**18% | "keep"**

**14% | "safe"**

**11% | "follow"**

**08% | "#blacklivesmatter"**

**07% | "send"**

**06% | "instagram"**

**05% | "delivery"**

**05% | "relief"**

**2019 Percentage: 40.72% 2020 Percentage: 59.28% Percentage Increase: 45.58%**

**____________________________________**

**Topic #19:**

**61% | "people"**

**06% | "benefit"**

**05% | "many"**

**2019 Percentage: 41.71% 2020 Percentage: 58.29% Percentage Increase: 39.74%**

**____________________________________**

**Topic #34:**

**39% | "know"**

**15% | "advocate"**

**10% | "push"**

**07% | "essential"**

**06% | "response"**

**2019 Percentage: 42.35% 2020 Percentage: 57.65% Percentage Increase: 36.15%**

**____________________________________**

**Topic #76:**

**28% | "ensure"**

**13% | "stay"**

**11% | "race"**

**08% | "understand"**

**08% | "equity"**

**08% | "bad"**

**06% | "million"**

**2019 Percentage: 42.86% 2020 Percentage: 57.14% Percentage Increase: 33.30%**

**____________________________________**

**Topic #49:**

**42% | "right"**

**16% | "healthcare"**

**06% | "view"**

**05% | "next"**

**2019 Percentage: 43.19% 2020 Percentage: 56.81% Percentage Increase: 31.53%**

**____________________________________**

**Topic #91:**

**29% | "part"**

**20% | "system"**

**12% | "listen"**

**10% | "free"**

**08% | "due"**

**05% | "tune"**

**05% | "allow"**

**2019 Percentage: 43.38% 2020 Percentage: 56.62% Percentage Increase: 30.54%**

**____________________________________**

**Topic #102:**

**28% | "worker"**

**17% | "action"**

**08% | "infant"**

**08% | "real"**

**07% | "play"**

**05% | "date"**

**05% | "role"**

**05% | "south"**

**2019 Percentage: 43.47% 2020 Percentage: 56.53% Percentage Increase: 30.07%**

**____________________________________**

**Topic #47:**

**19% | "home"**

**11% | "likely"**

**11% | "fund"**

**08% | "time"**

**06% | "july"**

**06% | "case"**

**05% | "nyc"**

**2019 Percentage: 43.50% 2020 Percentage: 56.50% Percentage Increase: 29.89%**

**____________________________________**

**Topic #35:**

**24% | "fight"**

**13% | "urge"**

**10% | "act"**

**10% | "reproductive"**

**05% | "cut"**

**2019 Percentage: 43.73% 2020 Percentage: 56.27% Percentage Increase: 28.69%**

**____________________________________**

**Topic #72:**

**14% | "racism"**

**09% | "associate"**

**08% | "base"**

**08% | "tool"**

**06% | "contact"**

**2019 Percentage: 44.52% 2020 Percentage: 55.48% Percentage Increase: 24.60%**

**____________________________________**

**Topic #15:**

**34% | "share"**

**30% | "story"**

**13% | "hear"**

**2019 Percentage: 44.68% 2020 Percentage: 55.32% Percentage Increase: 23.82%**

**____________________________________**

**Topic #51:**

**31% | "health"**

**19% | "maternal"**

**16% | "black"**

**05% | "address"**

**2019 Percentage: 44.81% 2020 Percentage: 55.19% Percentage Increase: 23.15%**

**____________________________________**

**Topic #25:**

**36% | "care"**

**12% | "video"**

**12% | "health"**

**08% | "access"**

**08% | "provider"**

**2019 Percentage: 44.85% 2020 Percentage: 55.15% Percentage Increase: 22.99%**

**____________________________________**

**Topic #57:**

**13% | "vote"**

**08% | "donate"**

**07% | "depend"**

**06% | "deal"**

**06% | "current"**

**06% | "favorite"**

**05% | "@fwhc"**

**04% | "bold"**

**2019 Percentage: 44.93% 2020 Percentage: 55.07% Percentage Increase: 22.56%**

**____________________________________**

**Topic #46:**

**65% | "thank"**

**10% | "join"**

**05% | "labor"**

**2019 Percentage: 45.11% 2020 Percentage: 54.89% Percentage Increase: 21.67%**

**____________________________________**

**Topic #68:**

**24% | "continue"**

**17% | "visit"**

**14% | "public"**

**10% | "control"**

**06% | "long"**

**05% | "expert"**

**05% | "brown"**

**2019 Percentage: 45.16% 2020 Percentage: 54.84% Percentage Increase: 21.41%**

**____________________________________**

**Topic #37:**

**58% | "need"**

**09% | "pregnancy"**

**08% | "postpartum"**

**2019 Percentage: 45.51% 2020 Percentage: 54.49% Percentage Increase: 19.74%**

**____________________________________**

**Topic #30:**

**33% | "community"**

**19% | "color"**

**14% | "celebrate"**

**10% | "provide"**

**2019 Percentage: 45.55% 2020 Percentage: 54.45% Percentage Increase: 19.52%**

**____________________________________**

**Topic #12:**

**32% | "#womenshistorymonth"**

**06% | "moment"**

**05% | "forget"**

**2019 Percentage: 45.68% 2020 Percentage: 54.32% Percentage Increase: 18.92%**

**____________________________________**

**Topic #48:**

**42% | "must"**

**09% | "challenge"**

**08% | "fear"**

**06% | "break"**

**05% | "co"**

**05% | "choice"**

**05% | "governor"**

**2019 Percentage: 46.08% 2020 Percentage: 53.92% Percentage Increase: 17.04%**

**____________________________________**

**Topic #97:**

**16% | "month"**

**10% | "practice"**

**09% | "enough"**

**08% | "clear"**

**08% | "count"**

**07% | "fail"**

**06% | "june"**

**05% | "willing"**

**05% | "documentary"**

**2019 Percentage: 46.14% 2020 Percentage: 53.86% Percentage Increase: 16.73%**

**____________________________________**

**Topic #56:**

**13% | "center"**

**13% | "pm"**

**12% | "die"**

**08% | "rate"**

**07% | "black"**

**07% | "midwife"**

**06% | "childbirth"**

**06% | "detention"**

**05% | "advocacy"**

**2019 Percentage: 46.38% 2020 Percentage: 53.62% Percentage Increase: 15.59%**

**____________________________________**

**Topic #11:**

**30% | "experience"**

**15% | "effort"**

**15% | "reproductive_justice"**

**06% | "episode"**

**05% | "guest"**

**2019 Percentage: 46.64% 2020 Percentage: 53.36% Percentage Increase: 14.40%**

**____________________________________**

**Topic #70:**

**35% | "live"**

**10% | "watch"**

**09% | "matter"**

**08% | "often"**

**05% | "last"**

**2019 Percentage: 46.99% 2020 Percentage: 53.01% Percentage Increase: 12.81%**

**____________________________________**

**Topic #98:**

**26% | "impact"**

**15% | "face"**

**12% | "could"**

**11% | "barrier"**

**10% | "rsvp"**

**05% | "news"**

**2019 Percentage: 47.00% 2020 Percentage: 53.00% Percentage Increase: 12.78%**

**____________________________________**

**Topic #74:**

**19% | "#advancingblackbirth"**

**16% | "proud"**

**15% | "partner"**

**10% | "join"**

**05% | "rights"**

**05% | "shift"**

**2019 Percentage: 47.07% 2020 Percentage: 52.93% Percentage Increase: 12.43%**

**____________________________________**

**Topic #18:**

**19% | "back"**

**18% | "world"**

**11% | "nee"**

**09% | "beautiful"**

**07% | "lift"**

**2019 Percentage: 47.37% 2020 Percentage: 52.63% Percentage Increase: 11.10%**

**____________________________________**

**Topic #108:**

**25% | "happen"**

**14% | "friday"**

**12% | "risk"**

**2019 Percentage: 47.45% 2020 Percentage: 52.55% Percentage Increase: 10.76%**

**____________________________________**

**Topic #10:**

**28% | "abortion"**

**12% | "bill"**

**07% | "pass"**

**06% | "organization"**

**05% | "lawmaker"**

**2019 Percentage: 47.47% 2020 Percentage: 52.53% Percentage Increase: 10.67%**

**____________________________________**

**Topic #63:**

**34% | "go"**

**14% | "still"**

**13% | "stand"**

**2019 Percentage: 47.62% 2020 Percentage: 52.38% Percentage Increase: 9.99%**

**____________________________________**

**Topic #16:**

**20% | "black"**

**16% | "mamas"**

**07% | "mama"**

**06% | "uplift"**

**06% | "jail"**

**2019 Percentage: 47.71% 2020 Percentage: 52.29% Percentage Increase: 9.59%**

**____________________________________**

**Topic #52:**

**28% | "let"**

**15% | "resource"**

**14% | "start"**

**08% | "s"**

**05% | "thrive"**

**2019 Percentage: 47.76% 2020 Percentage: 52.24% Percentage Increase: 9.37%**

**____________________________________**

**Topic #87:**

**33% | "day"**

**26% | "mother"**

**07% | "research"**

**06% | "amazing"**

**05% | "huge"**

**2019 Percentage: 47.94% 2020 Percentage: 52.06% Percentage Increase: 8.59%**

**____________________________________**

**Topic #9 :**

**15% | "release"**

**13% | "deserve"**

**08% | "incarcerate"**

**08% | "social_media"**

**07% | "result"**

**2019 Percentage: 47.98% 2020 Percentage: 52.02% Percentage Increase: 8.43%**

**____________________________________**

**Topic #17:**

**24% | "justice"**

**19% | "issue"**

**10% | "doula"**

**09% | "city"**

**06% | "new_york"**

**2019 Percentage: 48.00% 2020 Percentage: 52.00% Percentage Increase: 8.33%**

**____________________________________**

**Topic #0 :**

**11% | "april"**

**10% | "honor"**

**08% | "introduce"**

**07% | "wait"**

**06% | "legislation"**

**06% | "nc"**

**05% | "senator"**

**05% | "funding"**

**2019 Percentage: 48.00% 2020 Percentage: 52.00% Percentage Increase: 8.32%**

**____________________________________**

**Topic #65:**

**46% | "help"**

**07% | "meet"**

**07% | "virtual"**

**07% | "support"**

**05% | "@colorlatina"**

**05% | "#reprojustice"**

**2019 Percentage: 48.19% 2020 Percentage: 51.81% Percentage Increase: 7.50%**

**____________________________________**

**Topic #66:**

**32% | "use"**

**10% | "can"**

**10% | "dr"**

**08% | "place"**

**08% | "lot"**

**07% | "engage"**

**2019 Percentage: 48.47% 2020 Percentage: 51.53% Percentage Increase: 6.30%**

**____________________________________**

**Topic #78:**

**29% | "tell"**

**22% | "congress"**

**08% | "georgia"**

**05% | "ready"**

**2019 Percentage: 48.53% 2020 Percentage: 51.47% Percentage Increase: 6.08%**

**____________________________________**

**Topic #82:**

**21% | "demand"**

**13% | "high"**

**12% | "police"**

**09% | "force"**

**05% | "term"**

**2019 Percentage: 48.70% 2020 Percentage: 51.30% Percentage Increase: 5.32%**

**____________________________________**

**Topic #45:**

**24% | "show"**

**13% | "focus"**

**08% | "americans"**

**05% | "lack"**

**05% | "facility"**

**2019 Percentage: 48.86% 2020 Percentage: 51.14% Percentage Increase: 4.69%**

**____________________________________**

**Topic #69:**

**37% | "week"**

**10% | "plan"**

**09% | "health"**

**07% | "sign"**

**07% | "insurance"**

**05% | "cover"**

**2019 Percentage: 48.93% 2020 Percentage: 51.07% Percentage Increase: 4.36%**

**____________________________________**

**Topic #53:**

**35% | "protect"**

**20% | "school"**

**07% | "student"**

**05% | "wage"**

**04% | "value"**

**2019 Percentage: 49.11% 2020 Percentage: 50.89% Percentage Increase: 3.62%**

**____________________________________**

**Topic #32:**

**11% | "order"**

**09% | "midwifery"**

**08% | "man"**

**06% | "perinatal"**

**05% | "village"**

**05% | "teen"**

**05% | "inequity"**

**2019 Percentage: 49.12% 2020 Percentage: 50.88% Percentage Increase: 3.60%**

**____________________________________**

**Topic #3 :**

**24% | "important"**

**22% | "include"**

**15% | "pregnant"**

**07% | "consider"**

**04% | "participate"**

**2019 Percentage: 49.14% 2020 Percentage: 50.86% Percentage Increase: 3.52%**

**____________________________________**

**Topic #36:**

**37% | "life"**

**06% | "invite"**

**05% | "prevent"**

**05% | "kind"**

**04% | "judge"**

**04% | "trump"**

**2019 Percentage: 49.14% 2020 Percentage: 50.86% Percentage Increase: 3.49%**

**____________________________________**

**Topic #81:**

**34% | "today"**

**32% | "join"**

**08% | "together"**

**06% | "conversation"**

**2019 Percentage: 49.79% 2020 Percentage: 50.21% Percentage Increase: 0.83%**

**____________________________________**

**Topic #89:**

**12% | "#breastfeeding"**

**10% | "discrimination"**

**10% | "online"**

**08% | "combat"**

**07% | "dangerous"**

**06% | "statement"**

**2019 Percentage: 49.84% 2020 Percentage: 50.16% Percentage Increase: 0.62%**

**____________________________________**

**Topic #96:**

**22% | "#blackmaternalhealth"**

**14% | "#blackmamasmatter"**

**12% | "register"**

**07% | "webinar"**

**2019 Percentage: 49.97% 2020 Percentage: 50.03% Percentage Increase: 0.10%**

**____________________________________**

**Topic #104:**

**16% | "inspire"**

**13% | "future"**

**12% | "opportunity"**

**10% | "available"**

**07% | "color"**

**05% | "partnership"**

**2019 Percentage: 50.00% 2020 Percentage: 50.00% Percentage Increase: -0.01%**

**____________________________________**

**Topic #33:**

**28% | "read"**

**14% | "group"**

**10% | "receive"**

**08% | "book"**

**06% | "position"**

**2019 Percentage: 50.06% 2020 Percentage: 49.94% Percentage Increase: -0.23%**

**____________________________________**

**Topic #106:**

**46% | "make"**

**12% | "well"**

**10% | "always"**

**07% | "healthy"**

**2019 Percentage: 50.77% 2020 Percentage: 49.23% Percentage Increase: -3.04%**

**____________________________________**

**Topic #14:**

**30% | "state"**

**16% | "immigrant"**

**08% | "hold"**

**07% | "letter"**

**06% | "local"**

**2019 Percentage: 50.89% 2020 Percentage: 49.11% Percentage Increase: -3.48%**

**____________________________________**

**Topic #92:**

**43% | "get"**

**06% | "acknowledge"**

**05% | "sick"**

**2019 Percentage: 50.89% 2020 Percentage: 49.11% Percentage Increase: -3.49%**

**____________________________________**

**Topic #22:**

**14% | "monday"**

**10% | "reminder"**

**08% | "transgend"**

**07% | "board"**

**06% | "trans"**

**06% | "marginalize"**

**2019 Percentage: 51.07% 2020 Percentage: 48.93% Percentage Increase: -4.20%**

**____________________________________**

**Topic #55:**

**34% | "information"**

**09% | "campaign"**

**06% | "multiple"**

**06% | "deadline"**

**06% | "alternative"**

**05% | "commitment"**

**2019 Percentage: 51.21% 2020 Percentage: 48.79% Percentage Increase: -4.72%**

**____________________________________**

**Topic #5 :**

**18% | "give"**

**14% | "friend"**

**12% | "excited"**

**11% | "believe"**

**06% | "activist"**

**05% | "kill"**

**2019 Percentage: 51.55% 2020 Percentage: 48.45% Percentage Increase: -6.02%**

**____________________________________**

**Topic #95:**

**28% | "come"**

**16% | "person"**

**09% | "other"**

**05% | "rise"**

**05% | "early"**

**05% | "soon"**

**2019 Percentage: 51.75% 2020 Percentage: 48.25% Percentage Increase: -6.76%**

**____________________________________**

**Topic #84:**

**47% | "work"**

**20% | "learn"**

**06% | "job"**

**05% | "grateful"**

**2019 Percentage: 51.90% 2020 Percentage: 48.10% Percentage Increase: -7.32%**

**____________________________________**

**Topic #27:**

**12% | "drive"**

**11% | "take_action"**

**09% | "hour"**

**09% | "imperative"**

**06% | "political"**

**06% | "guide"**

**2019 Percentage: 52.08% 2020 Percentage: 47.92% Percentage Increase: -7.99%**

**____________________________________**

**Topic #54:**

**14% | "big"**

**10% | "business"**

**08% | "conference"**

**07% | "set"**

**06% | "healing"**

**06% | "prioritize"**

**06% | "possible"**

**06% | "goal"**

**05% | "rest"**

**2019 Percentage: 52.16% 2020 Percentage: 47.84% Percentage Increase: -8.27%**

**____________________________________**

**Topic #85:**

**11% | "honored"**

**10% | "training"**

**09% | "women"**

**08% | "staff"**

**07% | "roll"**

**06% | "alarm"**

**05% | "train"**

**2019 Percentage: 52.17% 2020 Percentage: 47.83% Percentage Increase: -8.33%**

**____________________________________**

**Topic #80:**

**25% | "call"**

**18% | "parent"**

**15% | "change"**

**09% | "house"**

**08% | "info"**

**06% | "attack"**

**2019 Percentage: 52.18% 2020 Percentage: 47.82% Percentage Increase: -8.35%**

**____________________________________**

**Topic #101:**

**47% | "woman"**

**28% | "black"**

**05% | "white"**

**2019 Percentage: 52.35% 2020 Percentage: 47.65% Percentage Increase: -8.98%**

**____________________________________**

**Topic #83:**

**24% | "say"**

**15% | "mental"**

**11% | "thing"**

**10% | "reduce"**

**05% | "aca"**

**2019 Percentage: 52.58% 2020 Percentage: 47.42% Percentage Increase: -9.81%**

**____________________________________**

**Topic #39:**

**23% | "policy"**

**16% | "leader"**

**11% | "leadership"**

**07% | "youth"**

**06% | "line"**

**2019 Percentage: 52.59% 2020 Percentage: 47.41% Percentage Increase: -9.84%**

**____________________________________**

**Topic #2 :**

**49% | "family"**

**10% | "program"**

**08% | "also"**

**05% | "support"**

**2019 Percentage: 52.80% 2020 Percentage: 47.20% Percentage Increase: -10.59%**

**____________________________________**

**Topic #7 :**

**17% | "be"**

**15% | "head"**

**09% | "chat"**

**07% | "ig_page"**

**06% | "#mentalhealth"**

**2019 Percentage: 52.88% 2020 Percentage: 47.12% Percentage Increase: -10.88%**

**____________________________________**

**Topic #60:**

**19% | "event"**

**07% | "protection"**

**06% | "collective"**

**06% | "process"**

**05% | "develop"**

**05% | "meet"**

**2019 Percentage: 52.88% 2020 Percentage: 47.12% Percentage Increase: -10.89%**

**____________________________________**

**Topic #77:**

**32% | "much"**

**14% | "importance"**

**08% | "answer"**

**08% | "test"**

**06% | "priority"**

**2019 Percentage: 52.91% 2020 Percentage: 47.09% Percentage Increase: -11.02%**

**____________________________________**

**Topic #1 :**

**16% | "list"**

**10% | "minute"**

**09% | "podcast"**

**07% | "jessica"**

**05% | "outcomes"**

**05% | "degree"**

**04% | "equality"**

**04% | "california"**

**2019 Percentage: 53.22% 2020 Percentage: 46.78% Percentage Increase: -12.10%**

**____________________________________**

**Topic #38:**

**12% | "violence"**

**11% | "seek"**

**08% | "trump_administration"**

**07% | "rule"**

**06% | "harm"**

**05% | "recently"**

**05% | "sweep"**

**2019 Percentage: 53.35% 2020 Percentage: 46.65% Percentage Increase: -12.57%**

**____________________________________**

**Topic #64:**

**30% | "see"**

**28% | "talk"**

**08% | "choose"**

**2019 Percentage: 53.50% 2020 Percentage: 46.50% Percentage Increase: -13.09%**

**____________________________________**

**Topic #99:**

**14% | "think"**

**09% | "federal"**

**08% | "racist"**

**07% | "example"**

**06% | "sure"**

**05% | "poor"**

**05% | "announce"**

**05% | "begin"**

**04% | "@nwlc"**

**2019 Percentage: 53.55% 2020 Percentage: 46.45% Percentage Increase: -13.26%**

**____________________________________**

**Topic #13:**

**25% | "end"**

**16% | "already"**

**10% | "service"**

**05% | "remember"**

**2019 Percentage: 54.06% 2020 Percentage: 45.94% Percentage Increase: -15.02%**

**____________________________________**

**Topic #105:**

**26% | "great"**

**08% | "#bwhi"**

**06% | "hiv_aids"**

**06% | "#onourownterms"**

**05% | "hiv"**

**04% | "stigma"**

**2019 Percentage: 54.14% 2020 Percentage: 45.86% Percentage Increase: -15.29%**

**____________________________________**

**Topic #43:**

**22% | "bring"**

**12% | "discussion"**

**09% | "breastfeed"**

**09% | "coalition"**

**06% | "#savingourmamas"**

**05% | "joy"**

**2019 Percentage: 54.33% 2020 Percentage: 45.67% Percentage Increase: -15.96%**

**____________________________________**

**Topic #50:**

**22% | "look"**

**18% | "speak"**

**09% | "name"**

**08% | "defend"**

**08% | "truth"**

**07% | "#mothersday"**

**05% | "politician"**

**2019 Percentage: 54.44% 2020 Percentage: 45.56% Percentage Increase: -16.30%**

**____________________________________**

**Topic #24:**

**24% | "tonight"**

**07% | "compare"**

**07% | "@mclemoremr"**

**07% | "gift"**

**2019 Percentage: 54.51% 2020 Percentage: 45.49% Percentage Increase: -16.53%**

**____________________________________**

**Topic #58:**

**28% | "kid"**

**16% | "young"**

**11% | "present"**

**09% | "offer"**

**06% | "charge"**

**06% | "message"**

**2019 Percentage: 54.56% 2020 Percentage: 45.44% Percentage Increase: -16.72%**

**____________________________________**

**Topic #61:**

**19% | "decision"**

**18% | "power"**

**14% | "girl"**

**07% | "strength"**

**06% | "income"**

**05% | "wellness"**

**2019 Percentage: 54.63% 2020 Percentage: 45.37% Percentage Increase: -16.96%**

**____________________________________**

**Topic #73:**

**21% | "country"**

**16% | "member"**

**12% | "national"**

**06% | "quality"**

**2019 Percentage: 54.77% 2020 Percentage: 45.23% Percentage Increase: -17.40%**

**____________________________________**

**Topic #23:**

**23% | "check"**

**14% | "voice"**

**13% | "first"**

**12% | "us"**

**06% | "powerful"**

**05% | "budget"**

**2019 Percentage: 54.83% 2020 Percentage: 45.17% Percentage Increase: -17.63%**

**____________________________________**

**Topic #62:**

**22% | "find"**

**21% | "way"**

**14% | "good"**

**08% | "question"**

**07% | "hard"**

**07% | "ask"**

**2019 Percentage: 54.94% 2020 Percentage: 45.06% Percentage Increase: -17.99%**

**____________________________________**

**Topic #75:**

**25% | "food"**

**10% | "tweetstorm"**

**07% | "breastfeeding"**

**06% | "blog_post"**

**06% | "investment"**

**04% | "#bmhw"**

**2019 Percentage: 55.31% 2020 Percentage: 44.69% Percentage Increase: -19.21%**

**____________________________________**

**Topic #107:**

**34% | "new"**

**16% | "put"**

**09% | "report"**

**07% | "education"**

**06% | "freedom"**

**05% | "number"**

**2019 Percentage: 55.48% 2020 Percentage: 44.52% Percentage Increase: -19.75%**

**____________________________________**

**Topic #21:**

**33% | "take"**

**18% | "lead"**

**09% | "stop"**

**06% | "serve"**

**05% | "#stopthebans"**

**2019 Percentage: 55.59% 2020 Percentage: 44.41% Percentage Increase: -20.10%**

**____________________________________**

**Topic #42:**

**23% | "baby"**

**12% | "connect"**

**11% | "increase"**

**10% | "hope"**

**08% | "never"**

**06% | "little"**

**2019 Percentage: 55.97% 2020 Percentage: 44.03% Percentage Increase: -21.33%**

**____________________________________**

**Topic #88:**

**27% | "would"**

**18% | "discuss"**

**14% | "able"**

**05% | "miss"**

**2019 Percentage: 56.04% 2020 Percentage: 43.96% Percentage Increase: -21.57%**

**____________________________________**

**Topic #100:**

**53% | "want"**

**13% | "self"**

**06% | "educator"**

**05% | "st"**

**2019 Percentage: 56.05% 2020 Percentage: 43.95% Percentage Increase: -21.60%**

**____________________________________**

**Topic #79:**

**34% | "child"**

**13% | "full"**

**08% | "spread"**

**08% | "raise"**

**08% | "word"**

**05% | "emergency"**

**2019 Percentage: 56.39% 2020 Percentage: 43.61% Percentage Increase: -22.68%**

**____________________________________**

**Topic #26:**

**14% | "affect"**

**14% | "law"**

**10% | "become"**

**09% | "tip"**

**07% | "piece"**

**06% | "government"**

**05% | "morning"**

**2019 Percentage: 56.69% 2020 Percentage: 43.31% Percentage Increase: -23.61%**

**____________________________________**

**Topic #67:**

**26% | "mom"**

**21% | "#maternaljustice"**

**09% | "hospital"**

**09% | "death"**

**09% | "patient"**

**04% | "team"**

**2019 Percentage: 57.15% 2020 Percentage: 42.85% Percentage Increase: -25.02%**

**____________________________________**

**Topic #6 :**

**18% | "@momsrising"**

**17% | "join"**

**16% | "pm_et"**

**08% | "@mamasconpoder"**

**07% | "leave"**

**07% | "tomorrow"**

**2019 Percentage: 57.88% 2020 Percentage: 42.12% Percentage Increase: -27.23%**

**____________________________________**

**Topic #93:**

**18% | "coverage"**

**15% | "medicaid"**

**13% | "affordable"**

**07% | "really"**

**06% | "#equalpay"**

**06% | "#protectourcare"**

**05% | "mark"**

**2019 Percentage: 58.05% 2020 Percentage: 41.95% Percentage Increase: -27.73%**

**____________________________________**

**Topic #94:**

**26% | "create"**

**13% | "feel"**

**12% | "space"**

**08% | "#trustblackwomen"**

**06% | "form"**

**06% | "ceo"**

**2019 Percentage: 58.28% 2020 Percentage: 41.72% Percentage Increase: -28.40%**

**____________________________________**

**Topic #40:**

**13% | "@repunderwood"**

**09% | "@repadams"**

**09% | "ill"**

**06% | "legacy"**

**05% | "administration"**

**05% | "setting"**

**2019 Percentage: 59.14% 2020 Percentage: 40.86% Percentage Increase: -30.90%**

**____________________________________**

**Topic #8 :**

**30% | "pay"**

**28% | "may"**

**11% | "fall"**

**2019 Percentage: 60.02% 2020 Percentage: 39.98% Percentage Increase: -33.40%**

**____________________________________**

**Topic #29:**

**25% | "year"**

**10% | "build"**

**07% | "welcome"**

**07% | "reposted"**

**06% | "history"**

**05% | "hand"**

**05% | "commit"**

**2019 Percentage: 61.22% 2020 Percentage: 38.78% Percentage Increase: -36.65%**

**____________________________________**

**Topic #20:**

**38% | "love"**

**16% | "#selfcare"**

**12% | "happy"**

**12% | "step"**

**2019 Percentage: 62.71% 2020 Percentage: 37.29% Percentage Increase: -40.53%**

**____________________________________**

**Topic #41:**

**12% | "@blkmamasmatter"**

**12% | "#blackmaternalhealthweek"**

**11% | "feature"**

**09% | "#blackmaternalhealth"**

**08% | "#bmhw19"**

**07% | "loved_one"**

**06% | "immediately"**

**05% | "#blackmamasmatter"**

**2019 Percentage: 62.72% 2020 Percentage: 37.28% Percentage Increase: -40.56%**

**____________________________________**

**Topic #44:**

**24% | "save"**

**16% | "money"**

**10% | "concern"**

**07% | "poverty"**

**07% | "intersection"**

**2019 Percentage: 67.75% 2020 Percentage: 32.25% Percentage Increase: -52.40%**

**____________________________________**

**K-Means Clustering**

In addition to topic modeling the tweets, we clustered tweets using a k-means model with *k* = 20, using a “bag-of-words” approach to utilize another methodology that could explain some of the themes in our corpus. In general, we found that topic modeling the tweets yielded clearer results of our corpus. However, there were some interesting results from this approach. One difference between topic modeling and k-means clustering is that the tweets analyzed by the former approach are included in multiple topics; in k-means, a tweet can only be in one cluster. So, for example, a tweet on Black Lives Matter (BLM) and public health could be included in multiple topics on BLM and public health, respectively.

For k-means clustering, our preprocessing step generated a list of all words appearing in the data, and then represented each tweet as a list of numbers indicating the count of each word appearing in the tweet. The k-means algorithm then groups tweets based on how similar these lists of counts are. The algorithm is not aware of individual word meanings and does not consider word order.

The reason we found that k-means was not as productive for our analysis as topic modeling is that, with every value of *k* we tried (that is, numbers of clusters), our algorithm outputted several clusters with very few (or one) tweet, and a single gigantic cluster with more than half our corpus. *k*=20 minimizes this effect, as shown below. This model assigns about 40% of tweets to cluster 0, with several moderately-sized clusters.


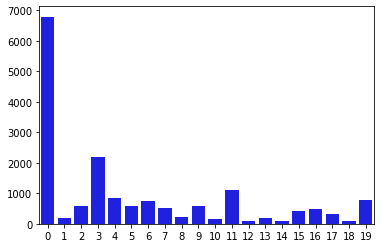


Figure 1: This chart shows how one cluster includes about half of the tweets. The rest are spread out across the rest of the clusters.

A few of the notable clusters are described below. These clusters focused on the following topics: healthcare and COVID-19, immigration and ICE, private prisons, abortion and reproductive justice, racial health disparities and pregnancy, and Black Maternal Health week. A word cloud of each of these clusters is listed below; the larger the word in the word cloud, the more significant that word is for the cluster. The clusters are ordered based on the topics that increased in 2020 to those that were more prevalent in 2019. We have included the topics that were more prevalent in 2019 because they demonstrate another impact of the pandemic: when organizations focused on Black maternal health need to focus on the pandemic, other concerns are less talked about. In this case, our research shows that those less talked about topics include conversations around Black Maternal Health week and private prisons.

###### *Cluster 1*

Discusses healthcare, Covid-19, community. The topic is more prevalent in 2020 than 2019.


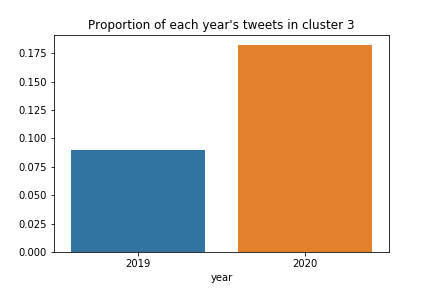


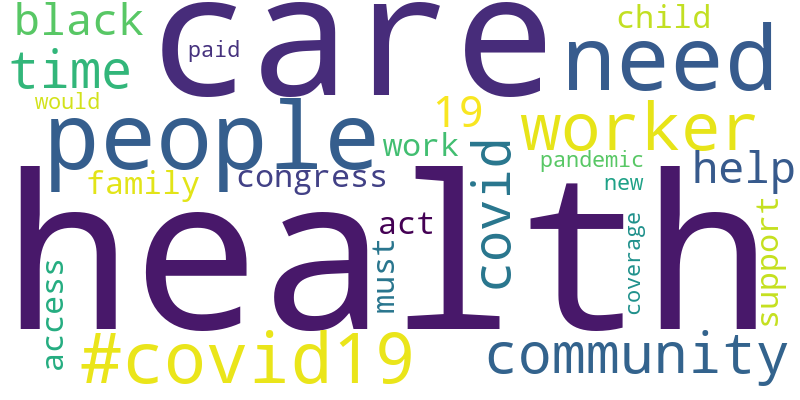


###### *Cluster 2*

This cluster is focused on abortion and reproductive justice. The topic is equally common in both years.


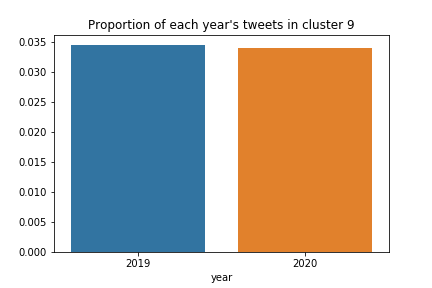


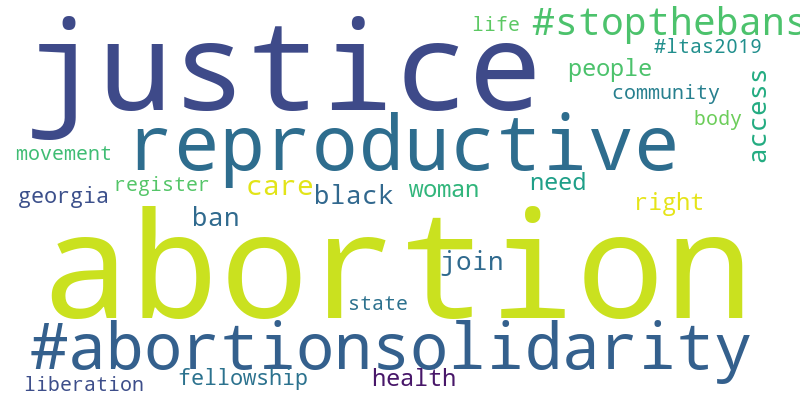


######

###### *Cluster 3*

Racial health disparities and pregnancy. The topic is discussed about the same in 2019 versus 2020.


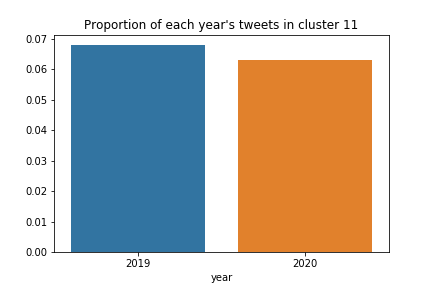


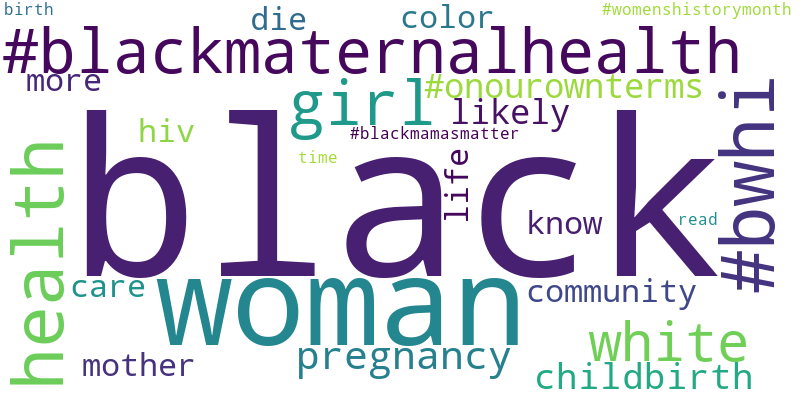

Supplement: Multimedia Appendix 1 [file infodemiology_v2i1e30885_app1.docx]
