# Supplementary figures and images for: US Black Maternal Health Advocacy Topics and Trends on Twitter: Temporal Infoveillance Study
Source: JMIR Infodemiology. 2022 Apr 20;2(1):e30885. doi: 10.2196/30885 (PMC9092478; doi:10.2196/30885)

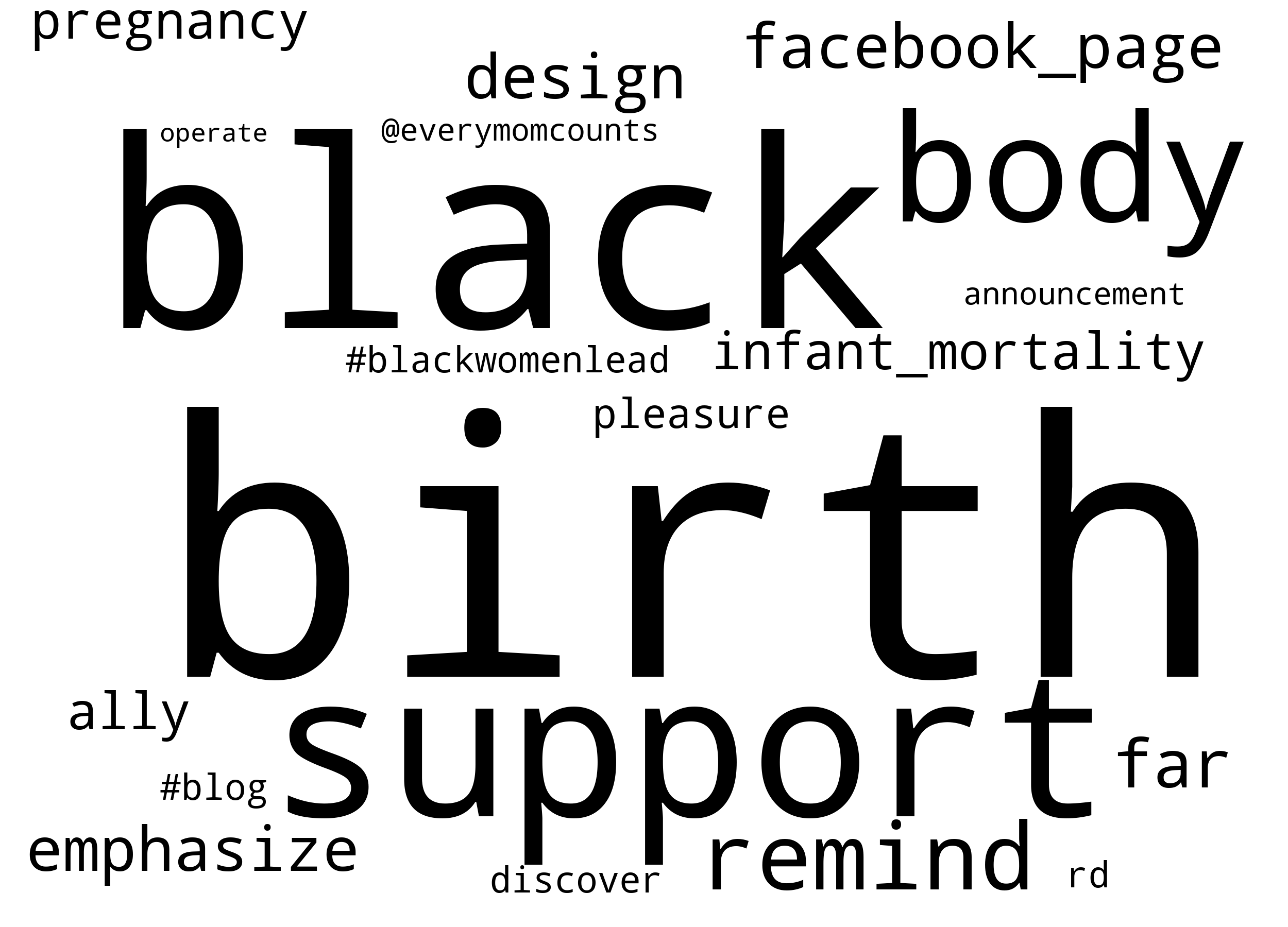

Supplement: Multimedia Appendix 2 [file infodemiology_v2i1e30885_app2.png]

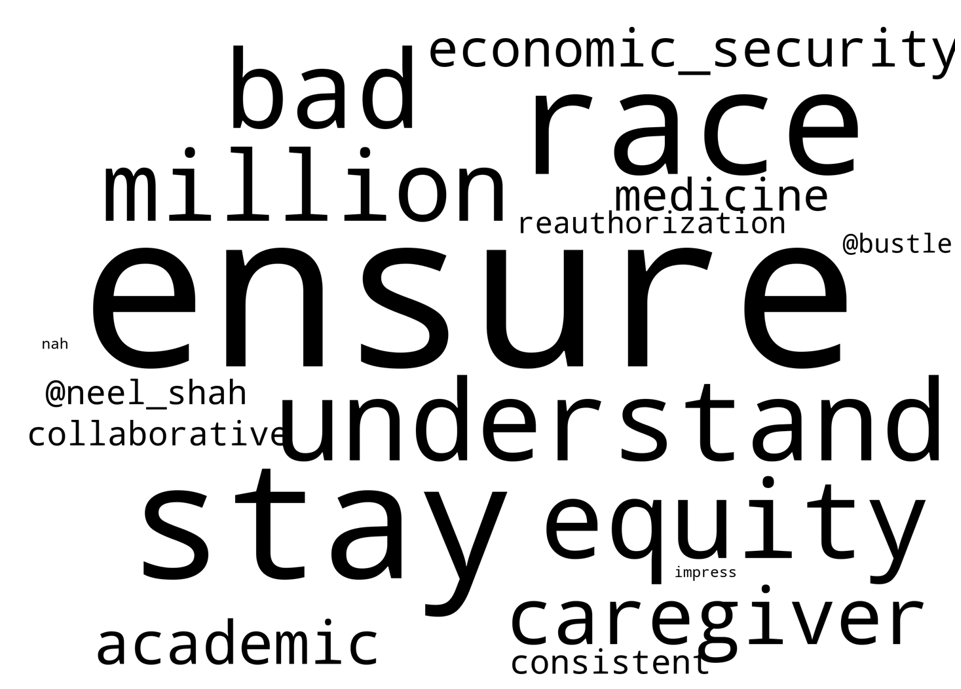

Supplement: Multimedia Appendix 3 [file infodemiology_v2i1e30885_app3.png]

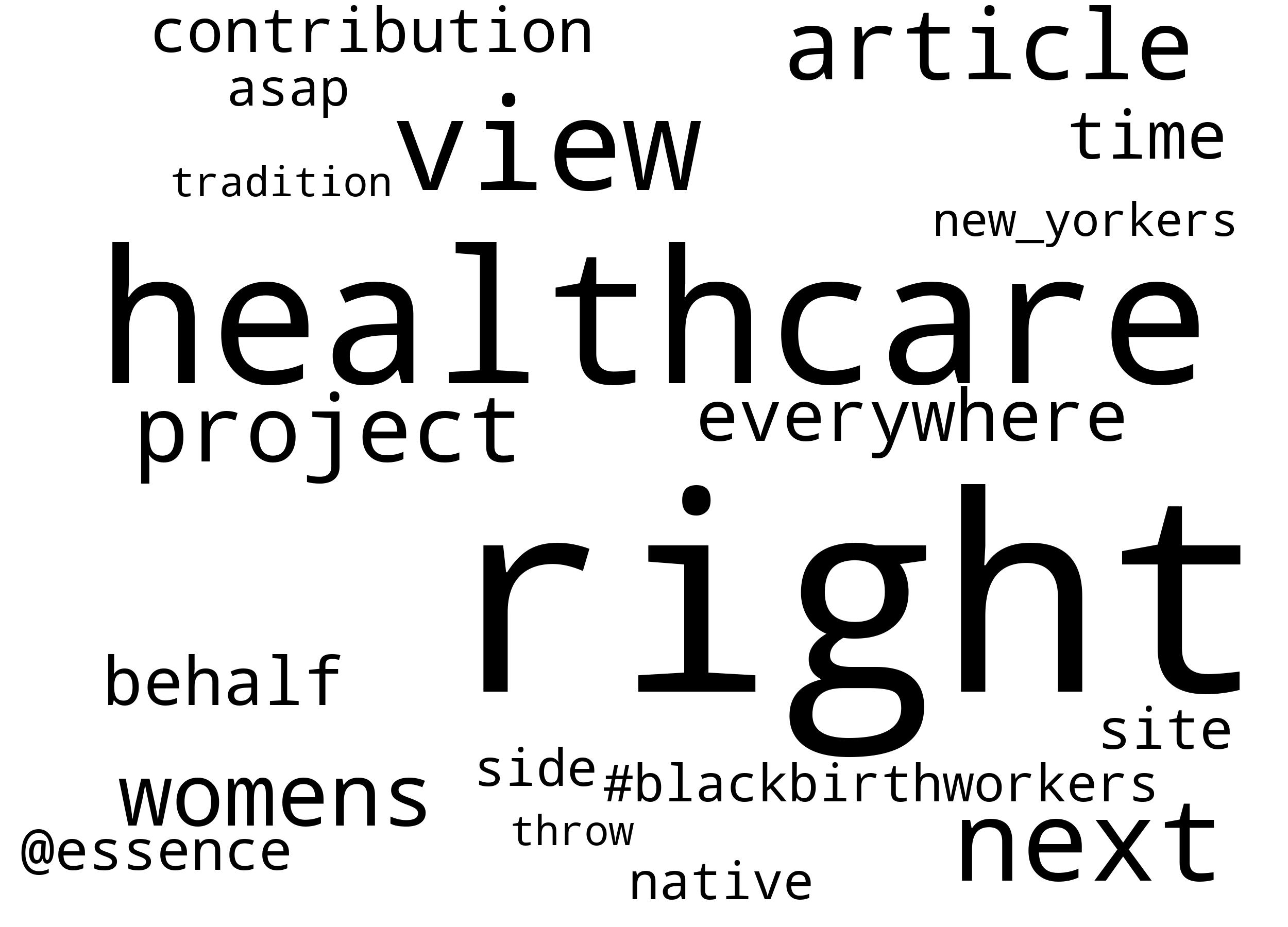

Supplement: Multimedia Appendix 4 [file infodemiology_v2i1e30885_app4.png]

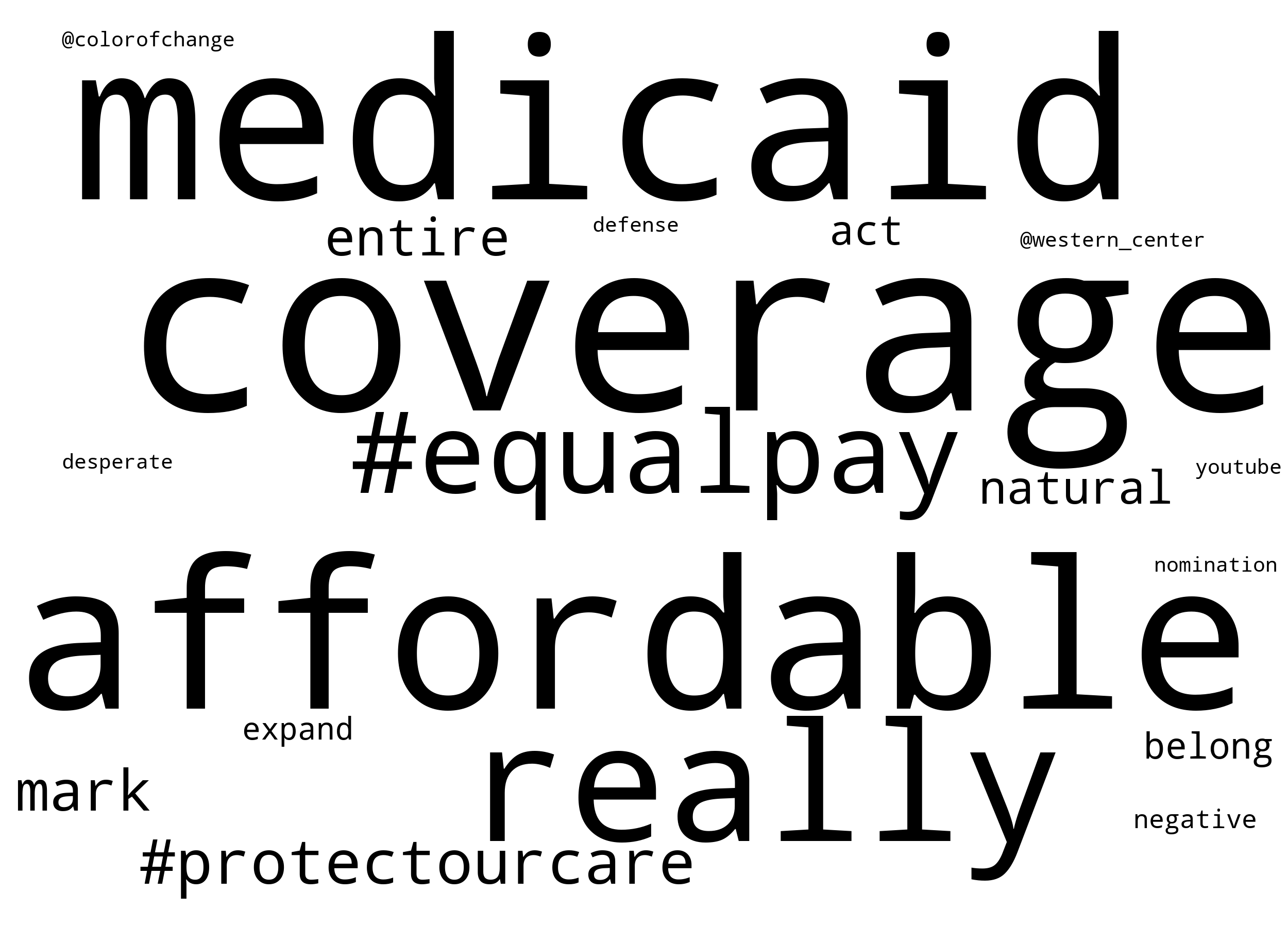

Supplement: Multimedia Appendix 5 [file infodemiology_v2i1e30885_app5.png]

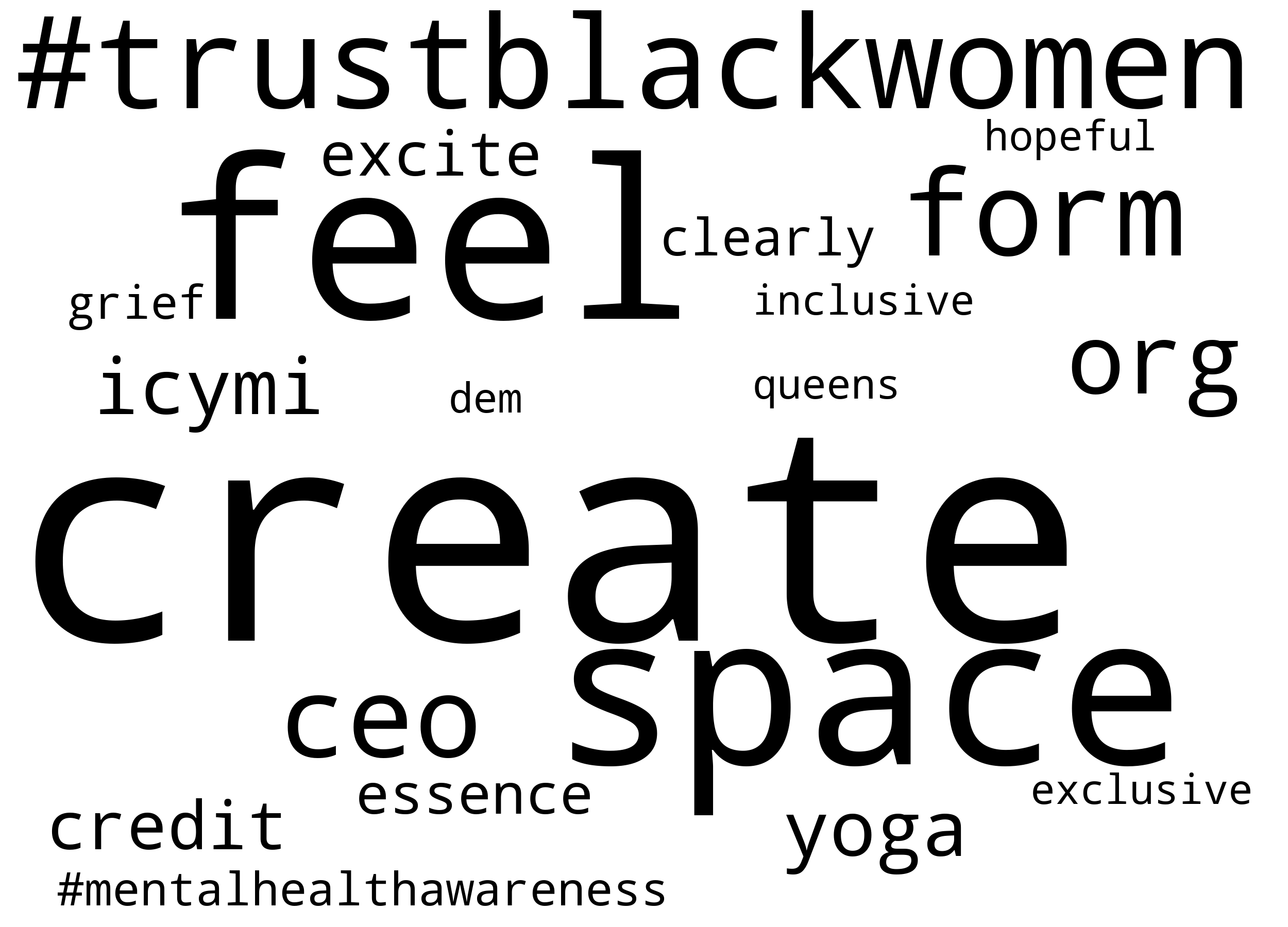

Supplement: Multimedia Appendix 6 [file infodemiology_v2i1e30885_app6.png]
